# Growing Neural PC-12 Cell on Crosslinked Silica Aerogels Increases Neurite Extension in the Presence of an Electric Field

**DOI:** 10.3390/jfb9020030

**Published:** 2018-04-20

**Authors:** Kyle J Lynch, Omar Skalli, Firouzeh Sabri

**Affiliations:** 1 Department of Physics and Materials Science, University of Memphis, Memphis, TN 38152, USA; kjlynch@memphis.edu; 2 Department of Biological Sciences, University of Memphis, Memphis, TN 38152, USA; oskalli@memphis.edu

**Keywords:** aerogel, porous, scaffold, substrate, PC-12, neuron, electrical stimulation, electric field, guidance

## Abstract

Externally applied electrical stimulation (ES) has been shown to enhance the nerve regeneration process and to influence the directionality of neurite outgrowth. In addition, the physical and chemical properties of the substrate used for nerve-cell regeneration is critical in fostering regeneration. Previously, we have shown that polyurea-crosslinked silica aerogels (PCSA) exert a positive influence on the extension of neurites by PC-12 cells, a cell-line model widely used to study neurite extension and electrical excitability. In this work, we have examined how an externally applied electric field (EF) influences the extension of neurites in PC-12 cells grown on two substrates: collagen-coated dishes versus collagen-coated crosslinked silica aerogels. The externally applied direct current (DC) bias was applied in vitro using a custom-designed chamber containing polydimethysiloxane (PDMS) embedded copper electrodes to create an electric field across the substrate for the cultured PC-12 cells. Results suggest orientation preference towards the anode, and, on average, longer neurites in the presence of the applied DC bias than with 0 V DC bias. In addition, neurite length was increased in cells grown on silica-crosslinked aerogel when compared to cells grown on regular petri-dishes. These results further support the notion that PCSA is a promising material for nerve regeneration.

## 1. Introduction

Aerogels are gaining attention as biomaterials because of their low bulk density, high surface area to volume ratio, high mechanical strength and ability to serve as a “host” to therapeutic molecules and cells [1,2]. Recent investigations of polymer-crosslinked silica aerogels (PCSA) have established their biocompatibility and biostability [3,4], the ability to detect them within the body using standard clinical imaging techniques [5,6,7], and their capacity to enhance the extension of neurites by PC-12 neural cells and dorsal root ganglia neurons when compared to conventional cell culture substrates [8,9,10]. The latter results suggest that crosslinked silica aerogels have the potential to enhance regeneration after nerve injury. Electrical stimulation (ES) in the form of electric fields (EF) is another method that has been proven to positively influence the extension of neurites by neural cells both in tissue-culture systems and in animal models [11,12,13,14,15,16]. This applies to both static and pulsed electric fields [17] which have been shown to accelerate neurite outgrowth in vitro [16,17,18,19], nerve regeneration in vivo [20,21,22,23], and in some cases influence the direction and orientation of neurite outgrowth [14]. Collectively, these studies suggest that the stimulation of severed nerves by electrical fields has the potential to enhance both nerve regeneration and the prospects of recovery after nerve injury [24]. 

The majority of the studies on the role of electric fields on neuronal cells have plated those cells on conductive substrates such as a tissue-culture plastic dish covered by polypyrole [25] or thin gold layers [26]. It remains to be seen whether electric fields also increase neurite length when neural cells are plated on PCSA. We have examined this possibility by using the neural cell line PC-12 cells and by examining how these cells respond to electric fields when placed on traditional tissue-culture substrates and on PCSA for comparison. PC-12 cells are a well-established model for peripheral neurons as they express neuronal markers such as the intermediate filament protein peripherin [27] and grow axon-like neurites in response to nerve growth factor (NGF) [28]. Moreover, these cells have been used extensively as a model to understand the influence of electrical stimulation [29,30,31,32]. Additionally, in our previous work [9], we have demonstrated the biocompatibility of PCSA and the ability of this material to foster the extension of neurites longer than those produced by PC-12 cells plated on conventional tissue culture substrates. In this work, the authors report, for the first time, the effect of direct current (DC) applied bias on PC-12 cells grown on PCSA as well as tissue-culture polystyrene (TCPS) serving as the control. The DC electrical stimulation was represented by an applied electrical field generated by a 15 V electrical potential difference. The influence of the applied electrical potential difference on PC-12 cells behavior was evaluated by monitoring (a) average neurite length, (b) directionality of neurite growth with respect to the location of the anode and cathode, and (c) neurite density. The results of three separate trials show that applied DC bias accelerates the growth rate of neurite extension for PC-12 cells plated on PCSA and that this difference was greater than for cells plated on TCPS. In addition, the preferred orientation and greater length of neurites toward the anode which was observed in previous studies for cells on TCPS was also found here for cells plated on PCSA.

## 2. Materials and Methods 

### 2.1. Aerogel Preparation 

Polyurea-crosslinked silica aerogel (PCSA) substrates were prepared by means of the sol-gel method and supercritical drying as described in detail previously [3,4]. The as-prepared samples were weighed and evaluated for integrity by means of optical microscopy prior to cell-culture steps. 

### 2.2. Electrostimulation Device 

**Figure 1:**
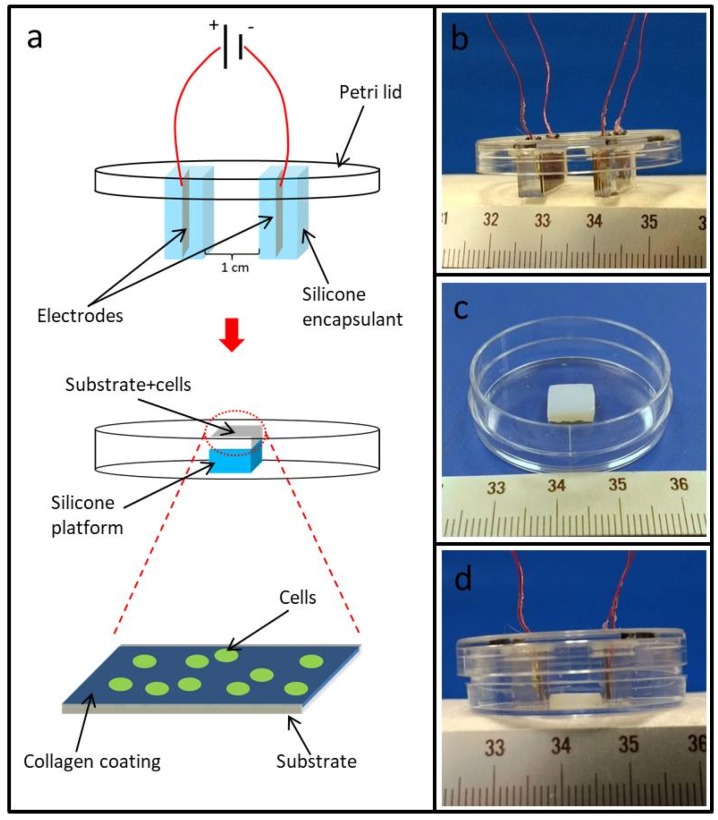
Electrical stimulation chamber: (**a**) schematic diagram of custom-built electrical stimulation chamber used for polyurea-crosslinked silica aerogel (PCSA) and tissue-culture polystyrene (TCPS) substrates. (**b**) Image of insulated electrodes and connecting leads affixed to a petri-dish lid. (**c**) PCSA substrate adhered to the bottom of a petri-dish. (**d**) Complete device with petri-dish lid placed over the bottom such that the electrodes fit snugly on opposite sides of the chosen substrate.

Electrodes used in this study were constructed in-house by soldering 30 AWG (American wire gauge) wires to two copper rectangular plates cut to the final desired size from a full sheet. The copper plates were then individually embedded in a uniform manner in Sylgard 184 silicone elastomer (Dow Corning) such that all sides of the copper plates were isolated from their surroundings with a 1 mm thick layer of Sylgard 184. Small holes were drilled into the lid of a 3.5 cm petri-dish and the wires were fed through the holes. Next the silicone-embedded copper plates (hereon referred to as electrodes) were attached to the interior of the lid with silicone adhesive (Permatex) 10 mm apart from one another (Figure 1a). PCSA or TCPS (control) substrates of (8 mm × 8 mm × 3 mm) (w × l × h) were then glued to the bottom of the petri dish with silicone adhesive (Figure 1b). The lid was then placed back on top of the petri-dish bottom now containing PCSA or TCPS substrates attached to the bottom. In its final form, when the lid was placed back on the petri-dish bottom, the two electrodes sat on either side of the PCSA or TCPS substrate extending beyond the limits of the sample (Figure 1c) such that the entire sample area was positioned within the electric field region. Prior to placing the lids back on the petri-dishes, PCSA and TCPS substrates were first coated with 4 µg/cm^2^ collagen (Invitrogen) (discussed in detail below) in a sterile biological hood prior to plating with cells (Figure 1d). All dimensions and distances were carefully selected and measured to ensure that the entire surface of the substrate (TCPS or PCSA) was uniformly exposed to the applied DC bias to the best of our ability. 

### 2.3. PC-12 Cell Culture

PC-12 pheochromocytoma cells obtained from the American Type Culture Collection (ATCC) were grown in Roswell Park Memorial Institute (RPMI) 1640 medium supplemented with GlutaMax, hydroxyethyl piperazineethanesulfonic acid (HEPES) buffer, 10% horse serum, 5% fetal bovine serum, and penicillin/streptomycin. Before each experiment, PC-12 cells plated on a collagen 1 coated TCPS petri-dish cells were “primed” for neural differentiation with 50 ng/mL NGF for 8 days in low serum medium (RPMI 1640 supplemented with GlutaMax, HEPES buffer, 1% horse serum, and penicillin/streptomycin). This medium was replaced every 2–3 days with fresh low serum medium with NGF. After 8 days, the cells were harvested from the dish and frozen in liquid nitrogen [33] until used for electrostimulation experiments. Details of the method have been described previously [9]. Both 3-day and 1-day cultures were prepared for PCSA and TCPS for comparison.

### 2.4. Collagen Coating of PC-12 Substrates 

8 mm × 8 mm × 3 mm (l × w × h) PCSA and TCPS substrates previously attached to the petri-dish bottom (Figure 1) were sterilized as previously described [3,4] through immersion in 100% ethanol followed by 1 h exposure to 254 nm ultraviolet (UV) light. Rat tail type I collagen (Invitrogen, Carlsbad, CA, USA) was diluted to a final concentration of 0.05 mg/mL in 20 mM acetic acid [9]. Collagen solution was then laid on the substrate to reach a protein density of 4 µg/cm^2^ and was allowed to sit for 1 h at room temperature in a sterile cabinet. Next, the collagen solution was removed with a vacuum line, and the samples were rinsed twice with serum free RPMI 1640.

### 2.5. Electrical Stimulation 

NGF-primed PC-12 cells were plated on the collagen I coated TCPS and PCSA electrical devices at a density of 1–1.5 × 10^4^ cells/cm^2^ in the presence of low serum medium containing 50 ng/mL NGF. Samples that were to be electrically stimulated were connected to a calibrated voltage source (Korad). The voltage source was set to the desired voltage setting and calibrated prior to commencing the experiment. Cells were plated on each substrate type and allowed to grow for 24 h, in the presence of an applied DC bias set to 15 V. The DC voltage was applied to the substrates containing cells approximately 3 h after the cell-plating stage. Cells plated on TCPS and PCSA devices with no applied potential source (setting at 0 V) served as a control. No electrophoresis was observed at 15 V as judged by the lack of bubble formation in the medium. For each condition, three separate trials were performed and results were expressed as means ± SEM (standard error of the mean).

### 2.6. COMSOL Computation of Electric Field 

The magnitude of the electric field over the substrates was modeled by using the equations which describe the relations between the electric field E with the gradient of the potential V and the divergence of the displacement field D with the free-charge density ρ_f_. Finite element analysis (FEA) was performed using COMSOL Multiphysics Modeling software version 5.1. A 2-D model was created consisting of 2 rectangles representing the copper anode and cathode at a distance of 10 mm apart encapsulated by larger rectangles representing Sylgard 184 silicone with a relative permittivity of 2.72 (Dow Corning, Midland, MI, USA). A 3.5 cm diameter circle, encircling the encapsulated electrodes was produced to mimic a petri-dish containing cell-culture medium. The cell-culture medium was assigned a relative permittivity of 80 [34]. After setting a potential difference of 15 V on the copper electrodes, a time independent study was initiated. A maximum mesh size of 3.5 × 10^−4^ and an absolute tolerance of 1 × 10^−6^ were used for this study.

### 2.7. Scanning Electron Microscopy (SEM)

Samples were fixed in 2.5% glutaraldehyde (Tousimis, Rockville, MD, USA) in 0.1 M sodium cacodylate buffer for 2 h followed by 2 × 10 min washes with 0.1 M sodium cacodylate buffer. Samples were then immersed in an aqueous solution of 1% osmium tetroxide (Electron Microscopy Sciences, Hatfield, PA, USA) for 1 h followed by 2 × 10 min washes in sodium cacodylate buffer and then dehydrated by a series of 10 min each washes in 10%, 30%, 50%, 70%, 90%, and 100% ethanol. Finally, all substrates were allowed to fully air dry before sputter deposition of 10 nm gold/palladium film and imaging with Nova NanoSEM 650 Field Emission Scanning Electron Microscope (FEI Co., Hillsboro, OR, USA).

### 2.8. Neurite Measurement

**Figure 2:**
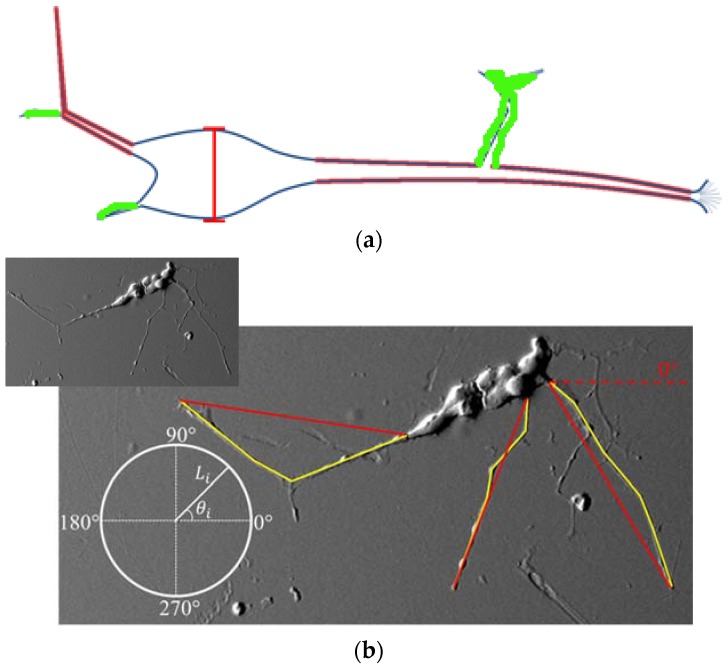
Neurite length measurement: (**a**) neurites were defined as the longest cytoplasmic extensions (outlined in red) originating from the cell body and short branches (outlined in green) originating from these extensions were not considered for measurement. (**b**) Diagram showing how measurements were performed with Image J yellow lines showing traced neurites and red lines representing the neurite end-to-end vector used to obtain the orientation with respect to a 0° reference angle. The anode was located at 270° while the cathode was located at 90° for all experiments. Insert shows the scanning electron microscope (SEM) image without Image J traces superimposed.

Neurite lengths and angles were measured using NIH Image J software. Neurite selection was performed as described in detail in previous published work by the authors [9]. The overlaid scale bar from scanning electron microscope (SEM) images was used to calibrate the measurement tools for length measurement. Neurites were treated as vectors and as such were measured with a series of straight line segments using the line segment tool, as shown in Figure 2a where Image J traces have been superimposed on an SEM image. Neurites were defined as the longest cytoplasmic extensions (outlined in red) originating from the cell body and short branches (outlined in green) originating from these extensions were not considered for measurement. Yellow lines show traced neurites used for this study. If the neurite branched, only the longest branch was considered. For all experiments, the anode corresponded to 270° (a negative y value) and the cathode corresponded to 90° (a positive y value). In all cases the positive X-axis was set at 0, as indicated on the polar plot in Figure 2b inset. Angles were determined by drawing a line connecting the cell soma to the end of the neurite using the straight line tool creating a resultant vector. Neurite length and density were analyzed with respect to orientation. To produce a neurite density plot, the Rayleigh test of uniformity was used to assess the orientation of the neurites [35]. To determine a preferred direction of growth, vector components were calculated by the use of:

(1) $$x=\frac{1}{N}\overset{N}{\underset{i=1}{\sum }}L_{i}\cos\theta_{i}$$

(2) $$y=\frac{1}{N}\overset{N}{\underset{i=1}{\sum }}L_{i}\sin\theta_{i}$$

where *N* represents the total number of neurites measured per trial per sample, *L* represents the individual neurite length, and *θ* represents the individual neurite angle. Additionally, neurites were broken into 180° sections and labeled as cathodal (those orientated toward the cathode) and anodal (those orientated towards the anode). Length and density values were compared amongst these groupings.

### 2.9. Statistical Analysis 

Statistical analysis of neurite length, neurite density, and directional growth bias was performed using a two-tailed student’s *t*-test. Significance (*, **) is defined as *p* < 0.05, and *p* < 0.005, respectively. In all cases error bars represent standard error of mean.

## 3. Results

### 3.1. Polyurea-Crosslinked Silica Aerogel (PCSA) Surface Effects on PC-12 Neurite Length

**Figure 3:**
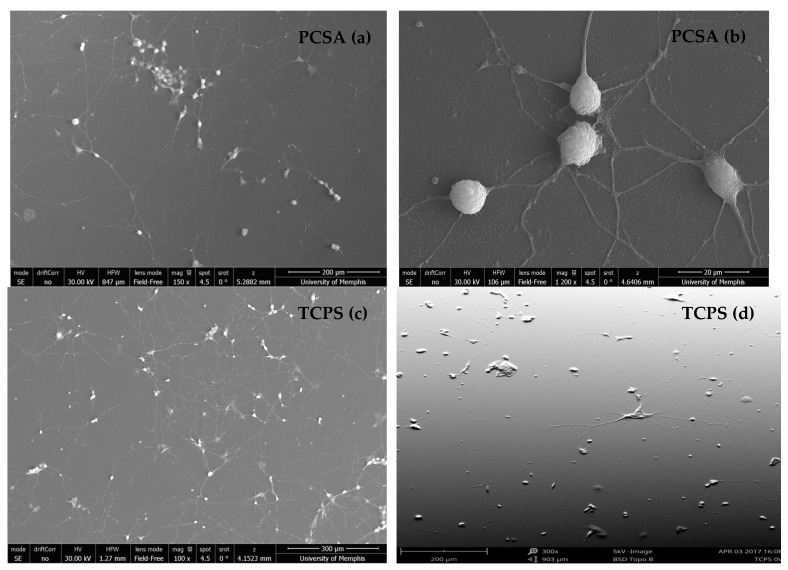
SEM images: SEM images of 0 V (unstimulated) PC12 cells—3-day culture: 3-day culture period of PC-12 cells grown with 0 V applied bias on collagen-coated (**a**), (**b**) PCSA and (**c**), (**d**) TCPS demonstrating the connectivity of extended neurites and the complexity of the growth pathways.

**Figure 4:**
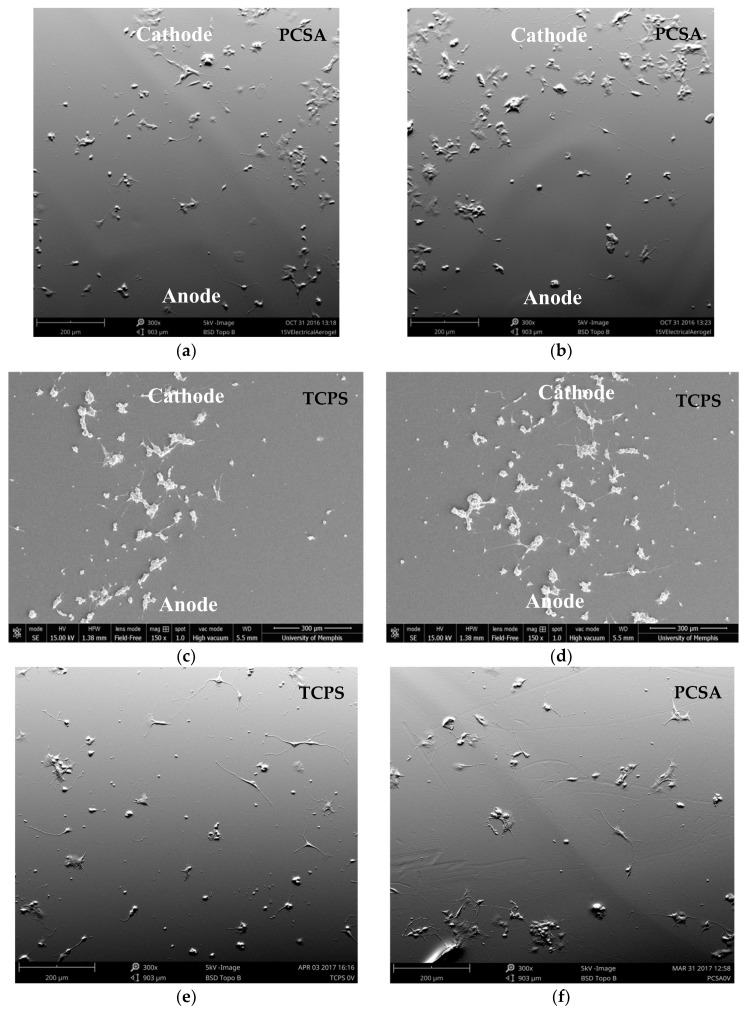
SEM images: 15 V 1-day culture: neurite extension in the presence of an applied bias of 15 V for a duration of 24 h on PCSA (**a**,**b**) and TCPS (**c**,**d**) taken randomly from different locations of the three separate trials for each type of substrate. The orientation of the anode and cathode has been indicated on each image. (**e**,**f**) show representative behavior of PC-12 cells on TCPS and PCSA at 0 V 1-day time point for comparison.

The effect of the culture period on neurite density and proliferation was compared for both substrates and shown in Figure 3. Figure 3 shows a 3-day culture period on collagen-coated PCSA (Figure 3a,b) and TCPS (Figure 3c,d) in the absence of applied bias. For both substrates, cells demonstrated neurite outgrowth. The large number and density of neurites for a 3-day culture made it challenging to distinguish the origin and growth cone of neurites, thereby hampering length measurements. Reducing the number of days in culture from 3 to 1 substantially reduced the connectivity of neurites, thereby enabling reliable length measurements. Hence, the electrical stimulation study was performed on 1-day cultures only and select SEM images from different locations are shown in Figure 4.

### 3.2. Electric Stimulation Effect on Neurite Length, Density, and Orientation 

Neurite length: Figure 4a,b show the response of PC-12 cells plated on PCSA to a 24 h-long continuous 15 V applied DC bias, collected from random locations. The orientation of the anode/cathode has been indicated on the images. The same conditions were used for PC-12 cells plated on TCPS (control) (Figure 4c,d). For comparison, SEM images from 0 V-1 Day cultures for both TCPS (Figure 4e) and PCSA (Figure 4d) substrates have also been presented in Figure 4.

**Figure 5:**
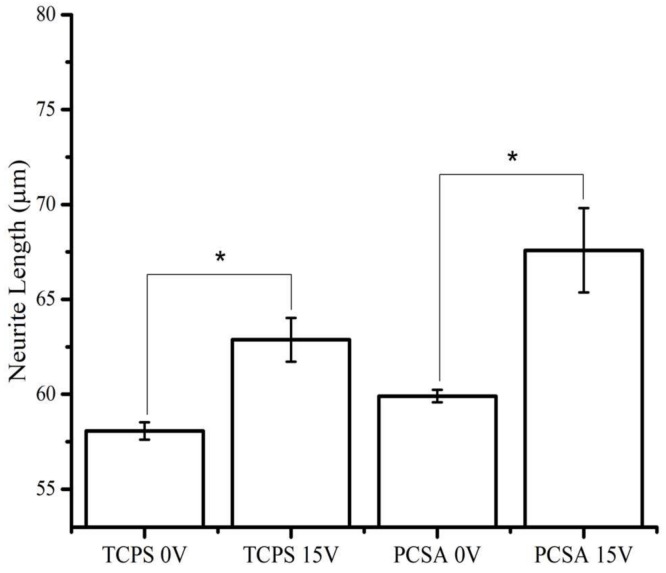
Effect of applied direct current (DC) bias and substrate on neurite length: effect of 15 V applied DC bias on PCSA and TCPS showing that the length of processes on aerogels is longer in the presence of a 24 h DC bias. Data averaged over 3 separate trials (sample size *n* = 3). Error bars show the standard error of mean and * represents p test values. Comparison of average length of all PC-12 neurites grown with and without the presence of an external DC-biased electric field (EF) grown on TCPS and PCSA substrates.

The analysis of 3 separate trials (*n* = 3) for each substrate at 0 V and at 15 V has been summarized and presented in Figure 5. The effect of applied DC bias was first analyzed without consideration of neurite orientation. The graph presented in Figure 5 shows that, at 0 V, neurites of cells plated on PCSA are about 10% longer that those extended by cells plated on TCPS. For both cells plated on TCPS and PCSA, 15 V externally applied electrical stimulation increased neurite length by at least ~12%. Because of the cumulative effects of substrate and of electrical stimulation, at 15 V cells plated on PCSA have extended neurites that are about 10% greater than cells plated on TCPS and subjected to the same electrical stimulation. Also, a qualitative assessment of the separate trials showed no migration of cell body for either substrate at the 15 V applied bias. 

**Figure 6:**
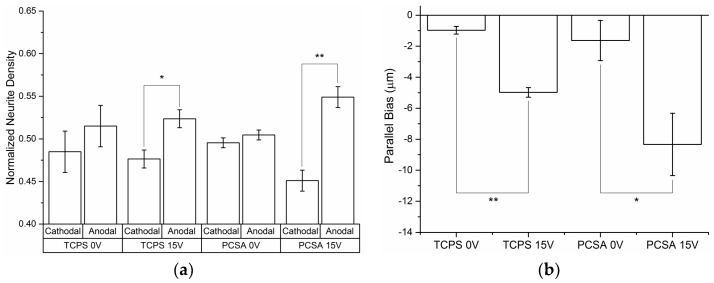
Effect of electrical stimulation (ES) on neurite density and orientation: (**a**) all neurites were summed as vectors indicating the bias or the preferred direction of growth on TCPS and PCSA with and without ES. (**b**) Average length of neurites in the anodal- and cathodal-facing groups of PC-12 neurites growing in the presence of an external EF on TCPS and PCSA substrates.

Neurite density: PC-12 neurites were divided into two 180° sections, anodal- and cathodal-facing (described in Section 2.8) for determining the neurite orientations. Neurites in the anodal- and cathodal-facing groups were then counted and normalized for the total of number of extensions counted per treatment group. Figure 6a compares the neurite counts for each treatment group. Electrically stimulated samples showed a significant increase in the number of neurites growing toward the anode compared to the cathodal-facing group. However, 0 V treatment showed no statistically significant orientation preference for either substrate. Neurites orientated towards the anode on average were longer than those growing towards the cathode.

### 3.3. Directional Bias

Treating the neurites in each group as vectors, the neurites were vector summed in accordance with relationships 1 and 2 and projected perpendicular to the field direction (Lcosθ) or, parallel to the field direction (Lsinθ). Figure 6b shows the projection of neurites parallel to the externally applied electric field for both substrates, with the arrow indicating the field direction. 15 V groups experienced a significant bias towards the anode (negative y-direction), whereas the 0 V y- and x-components (not shown) of all groups showed no significant bias and no statistical difference between these growth rates. Composite tracings of the cells showed a roughly symmetrical pattern of growth along the x and y directions.

### 3.4. COMSOL Computation of Electric Field

**Figure 7:**
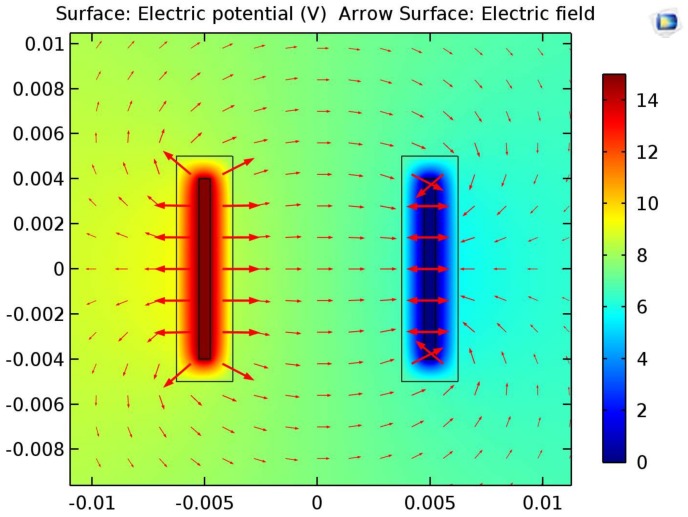
COMSOL: Vector field displaying the EF distribution generated by the electrical device. Vectors were plotted on a logarithmic scale for clarity.

To determine the strength of the electric field that cells were exposed to, a computational model was created with the use of COMSOL. A relatively uniform electric field of 210 mV/mm was computed within the cell-growth area between the two electrodes which agrees with hand calculations. Figure 7 shows the electric field distribution on a logarithmic scale.

## 4. Discussion

PC-12 cells are a good in vitro model for studying the mechanisms of neurite outgrowth and identifying factors influencing this process [36]. Since directed neurite outgrowth is important for nerve regeneration, PC-12 cells are also a useful model for nerve regeneration. Previously, we showed that, compared to TCPS, PCSA represents a substrate that enhances the length of neurites extended by PC-12 [9]. The presence of the collagen layer is not expected to alter the response of the cells to the combined effect of electrical stimulation and substrate. Based on the study published by Willitz et al. [37] collagen layers with the concentration used here have an insignificant stiffness which means that cells are in fact recognizing the different substrates and their influence is not masked by the collagen layer. Electric fields have also been shown to increase the length of neurites of cells grown on TCPS. Our results show that electric fields also enhance neurite outgrowth by PC-12 cells plated on PCSA when compared to cells plated on PCSA but not stimulated by an electric field. Compared to unstimulated cells grown on TCPS, neurites extended by PC-12 cells on electrically stimulated PCSA are about 20% longer, and about 10% longer than neurites extended by PC-12 cells on electrically stimulated TCPS. 

It was shown here that not only is the PCSA surface conducive to longer PC-12 neurite extension, but also the addition of externally-applied DC bias (of 200 mV/mm) selectively increased the growth rate of PC-12 neurites growing towards the anode rather than the cathode on both TCPS (control) and PCSA substrates. This corroborates previous work by Cork et al. [30] and Blumenthal et al. [38]. Under further analysis the data suggests that the external electric field stimulates longer extensions towards the anode, and does not appear to have a noticeable effect on extensions oriented towards the cathode. There was no statistically significant difference between TCPS and PCSA cathodal-facing neurite length and their respective 0 V controls. In addition to analyzing neurite length, neurite density in response to the electric field was also considered. An increase in the number of neurites facing the anode relative to the number of neurites facing the cathode was observed when considering the total number of neurites in the anodal and cathodal groups as a percentage, which also corroborates a previous study done by Cork et al. [30].

Endogenous electric fields of varying strengths occur naturally in vivo. With no injury present, endogenous field strengths are of the order of 0–10 mV/mm [39]. However, upon injury endogenous field strengths can increase 10-fold at the site of injury in mammalian tissue [26]. For example, in guinea pig spinal cord explants, endogenous field strengths of up to 200 mV/mm have been reported [40]. A potential difference of 15 V resulting in an EF strength of 200 mV/mm was chosen for this study because it is at the upper end of what is seen in mammalian tissue naturally and resulted in an EF strength comparable to those used in previous studies of this nature. The results indicate that it is possible to accelerate neurite growth and influence directionality. Previous studies have focused on conducting substrates such that a current is passed through the substrate [41]; the results presented here show similar behavior, but in the presence of an electric field applied to PCSA. Altogether, these results support the notion that PCSA is a promising material to support nerve regeneration as, alone, they enhance the outgrowth of neurites, while in the presence of an electric field this outgrowth is boosted and exhibits a definite polarity.

## References

[1] Maleki H., Durães L., Portugal A. (2014). An overview on silica aerogels synthesis and different mechanical reinforcing strategies. J. Non-Cryst. Solids.

[2] Hamedi M., Karabulut E., Marais A., Herland A., Nyström G., Wågberg L. (2013). Nanocellulose Aerogels Functionalized by Rapid Layer-by-Layer Assembly for High Charge Storage and Beyond. Angew. Chem. Int. Ed..

[3] Sabri F., Cole J.A., Scarbrough M.C., Leventis N. Investigation of crosslinked silica aerogels for implant applications. Proceedings of the 2011 Biomedical Sciences and Engineering Conference: Image Informatics and Analytics in Biomedicine.

[4] Sabri F., Boughter J.D., Gerth D., Skalli O., Phung T.C.N., Tamula G.R.M., Leventis N. (2012). Histological Evaluation of the Biocompatibility of Polyurea Crosslinked Silica Aerogel Implants in a Rat Model: A Pilot Study. PLoS ONE.

[5] Allison S.W., Baker E.S., Lynch K.J., Sabri F. (2015). In vivo X-ray imaging of phosphor-doped PDMS and phosphor-doped aerogel biomaterials. Int. J. Polym. Mater. Polym. Biomater..

[6] Sabri F., Sebelik M.E., Meacham R., Boughter J.D., Challis M.J., Leventis N. (2013). In Vivo Ultrasonic Detection of Polyurea Crosslinked Silica Aerogel Implants. PLoS ONE.

[7] Allison S.W., Baker E.S., Lynch K.J., Sabri F. (2017). In vivo X-Ray excited optical luminescence from phosphor-doped aerogel and Sylgard 184 composites. Radiat. Phys. Chem..

[8] Sabri F., Cole J.A., Scarbrough M.C., Leventis N. (2012). Investigation of Polyurea-Crosslinked Silica Aerogels as a Neuronal Scaffold: A Pilot Study. PLoS ONE.

[9] Lynch K.J., Skalli O., Sabri F. (2017). Investigation of surface topography and stiffness on adhesion and neurites extension of PC12 cells on crosslinked silica aerogel substrates. PLoS ONE.

[10] Sabri F., Gerth D., Tamula G.R., Phung T.C., Lynch K.J., Boughter J.D. (2014). Novel technique for repair of severed peripheral nerves in rats using polyurea crosslinked silica aerogel scaffold. J. Investig. Surg..

[11] Jaffe L.F., Stern C.D. (1979). Strong electrical currents leave the primitive streak of chick embryos. Science.

[12] Borgens R.B., Blight A.R., McGinnis M.E. (1990). Functional recovery after spinal cord hemisection in guinea pigs: The effects of applied electric fields. J. Comp. Neurol..

[13] Francis J.T., Gluckman B.J., Schiff S.J. (2003). Sensitivity of Neurons to Weak Electric Fields. J. Neurosci..

[14] Yao L., Shanley L., McCaig C., Zhao M. (2008). Small applied electric fields guide migration of hippocampal neurons. J. Cell. Physiol..

[15] McCaig C., Rajnicek A. (1991). Electrical fields, nerve growth and nerve regeneration. Exp. Physiol..

[16] Patel N., Poo M.M. (1982). Orientation of neurite growth by extracellular electric fields. J. Neurosci..

[17] Blackman C.F., Benane S.G., House D.E. (1993). Evidence for direct effect of magnetic fields on neurite outgrowth. FASEB J..

[18] Schmidt C.E., Shastri V.R., Vacanti J.P., Langer R. (1997). Stimulation of neurite outgrowth using an electrically conducting polymer. Proc. Natl. Acad. Sci. USA.

[19] Valentini R.F., Vargo T.G., Gardella J.A., Aebischer P. (1992). Electrically charged polymeric substrates enhance nerve fibre outgrowth in vitro. Biomaterials.

[20] Aebischer P., Valentini R.F., Dario P., Domenici C., Galletti P.M. (1987). Piezoelectric guidance channels enhance regeneration in the mouse sciatic nerve after axotomy. Brain Res..

[21] Valentini R.F., Sabatini A.M., Dario P., Aebischer P. (1989). Polymer electret guidance channels enhance peripheral nerve regeneration in mice. Brain Res..

[22] Sisken B.F., Kanje M., Lundborg G., Herbst E., Kurtz W. (1989). Stimulation of rat sciatic nerve regeneration with pulsed electromagnetic fields. Brain Res..

[23] Udina E., Furey M., Busch S., Silver J., Gordon T., Fouad K. (2008). Electrical stimulation of intact peripheral sensory axons in rats promotes outgrowth of their central projections. Exp. Neurol..

[24] Ghasemi-Mobarakeh L., Prabhakaran M.P., Morshed M., Nasr-Esfahani M.H., Baharvand H., Kiani S., Al-Deyab S.S., Ramakrishna S. (2011). Application of conductive polymers, scaffolds and electrical stimulation for nerve tissue engineering. J. Tissue Eng. Regen. Med..

[25] Gomez N., Lee J.Y., Nickels J.D., Schmidt C.E. (2007). Micropatterned Polypyrrole: A Combination of Electrical and Topographical Characteristics for the Stimulation of Cells. Adv. Funct. Mater..

[26] Park J.S., Park K., Moon H.T., Woo D.G., Yang H.N., Park K.-H. (2009). Electrical Pulsed Stimulation of Surfaces Homogeneously Coated with Gold Nanoparticles to Induce Neurite Outgrowth of PC12 Cells. Langmuir.

[27] Portier M.-M., Brachet P., Croizat B., Gros F. (1983). Regulation of Peripherin in Mouse Neuroblastoma and Rat PC 12 Pheochromocytoma Cell Lines. Dev. Neurosci..

[28] Levi A., Biocca S., Cattaneo A., Calissano P. (1988). The mode of action of nerve growth factor in PC12 cells. Mol. Neurobiol..

[29] Chang Y.-J., Hsu C.-M., Lin C.-H., Lu M.S.-C., Chen L. (2013). Electrical stimulation promotes nerve growth factor-induced neurite outgrowth and signaling. Biochim. Biophys. Acta BBA Gen. Subj..

[30] Cork R.J., McGinnis M.E., Tsai J., Robinson K.R. (1994). The growth of PC-12 neurites is biased towards the anode of an applied electrical field. J. Neurobiol..

[31] Manivannan S., Terakawa S. (1994). Rapid sprouting of filopodia in nerve terminals of chromaffin cells, PC12 cells, and dorsal root neurons induced by electrical stimulation. J. Neurosci..

[32] Zhang Z., Rouabhia M., Wang Z., Roberge C., Shi G., Roche P., Li J., Dao L.H. (2007). Electrically Conductive Biodegradable Polymer Composite for Nerve Regeneration: Electricity-Stimulated Neurite Outgrowth and Axon Regeneration. Artif. Organs.

[33] Rukenstein A., Greene L.A. (1983). The quantitative bioassay of nerve growth factor: Use of frozen ‘primed’ PC12 pheochromocytoma cells. Brain Res..

[34] Floriano W.B., Nascimento M.A. (2004). Dielectric constant and density of water as a function of pressure at constant temperature. Braz. J. Phys..

[35] Kapur T.A., Shoichet M.S. (2004). Immobilized concentration gradients of nerve growth factor guide neurite outgrowth. J. Biomed. Mater. Res. Part A.

[36] Kershner L., Welshhans K. (2017). RACK1 regulates neural development. Neural Regen. Res..

[37] Willits R.K., Skornia S.L. (2004). Effect of collagen gel stiffness on neurite extension. J. Biomater. Sci. Polym. Ed..

[38] Blumenthal N.R., Hermanson O., Heimrich B., Shastri V.P. (2014). Stochastic nanoroughness modulates neuron–astrocyte interactions and function via mechanosensing cation channels. Proc. Natl. Acad. Sci. USA.

[39] Baer M.L., Henderson S.C., Colello R.J. (2015). Elucidating the Role of Injury-Induced Electric Fields (EFs) in Regulating the Astrocytic Response to Injury in the Mammalian Central Nervous System. PLoS ONE.

[40] Messerli M.A., Graham D.M. (2011). Extracellular Electrical Fields Direct Wound Healing and Regeneration. Biol. Bull..

[41] Weng B., Liu X., Shepherd R., Wallace G.G. (2012). Inkjet printed polypyrrole/collagen scaffold: A combination of spatial control and electrical stimulation of PC12 cells. Synth. Met..

